# MicroRNAs implicated in canine diffuse large B‐cell lymphoma prognosis

**DOI:** 10.1002/2211-5463.13887

**Published:** 2024-09-01

**Authors:** Nelly O. Elshafie, Michael Gribskov, Nathanael I. Lichti, Ekramy E. Sayedahmed, Michael O. Childress, Andrea Pires dos Santos

**Affiliations:** ^1^ Department of Comparative Pathobiology Purdue University West Lafayette IN USA; ^2^ Department of Biological Sciences Purdue University West Lafayette IN USA; ^3^ Bindley Bioscience Center Purdue University West Lafayette IN USA; ^4^ Department of Veterinary Clinical Sciences Purdue University West Lafayette IN USA; ^5^ Purdue Institute for Cancer Research Purdue University West Lafayette IN USA

**Keywords:** cancer biomarkers, canine DLBCL, diffuse large B‐cell lymphoma, miRNAs, multicentric, sRNA sequencing

## Abstract

Diffuse large B‐cell lymphoma (DLBCL) is the most prevalent subtype of non‐Hodgkin lymphoma (NHL) in domestic dogs, with many similarities to its human counterpart. The progression of the disease is rapid, and treatment must be initiated early to achieve cancer remission and extend life. This study examined the relationship between progression‐free survival (PFS) and microRNA (miRNA) expression in dogs with DLBCL. miRNAs are small non‐coding RNA molecules that typically regulate gene expression post‐transcriptionally. They are involved in several pathophysiological processes, including the growth and progression of cancer. Based on the analysis of small RNA sequencing (sRNA‐seq) data, we validated a group of miRNAs in lymph nodes from 44 DLBCL‐affected dogs with known outcomes. We used quantitative PCR to quantify their expression and report a specific subset of miRNAs is associated with decreased PFS in dogs with DLBCL. The miR‐192‐5p and miR‐16‐5p expression were significantly downregulated in dogs with increased PFS. These results indicate that miRNA profiling may potentially identify dogs with DLBCL that will experience poor outcomes following treatment. Identifying specific miRNAs that correlate with the progression of canine DLBCL could aid the development of individualized treatment regimens for dogs.

## Graphical abstract

Our study explores how microRNAs (miRNAs) influence canine diffuse large B‐cell lymphoma (DLBCL) outcomes. We found specific miRNAs linked to shorter survival times, suggesting that miRNA profiling could help predict DLBCL prognosis. This research may lead to personalized dog treatments and be relevant for human lymphoma studies.

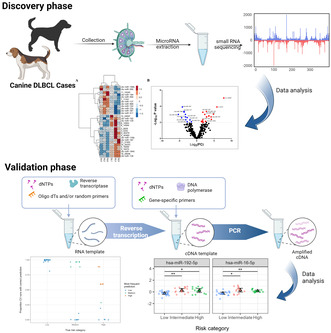

**Abbreviations**
ANOVA: analysis of variance
BOR: best overall response
cDNA: complementary DNA
CHOP: cyclophosphamide, doxorubicin, vincristine, and prednisone (chemotherapy regimen)
DEMs: differentially expressed miRNAs
DLBCL: diffuse large B‐cell lymphoma
FDR: false discovery rate
FFPE: formalin‐fixed paraffin‐embedded
IPC: inter‐plate calibrator
NHL: non‐Hodgkin lymphoma
PFS: progression‐free survival
RT‐qPCR: reverse transcription quantitative polymerase chain reaction
sRNA‐seq: small RNA sequencing
UV: ultraviolet

The most frequent form of lymphoma in dogs and humans is the diffuse large B‐cell lymphoma (DLBCL). It represents a wide range of diseases regarding morphology and subsequently, its outcomes are difficult to predict. Poor inter‐examiner consistency and limited success in outcome prediction are commonplace challenges with traditional morphologic subclassification [1]. Thus, developing and validating new prognostic tools is essential. In human studies, the International Prognostic Index (IPI) is the most crucial prognostic indicator in diffuse large B‐cell lymphoma cases in humans. All clinical trials use the IPI because of its reliability as a prognostic tool in DLBCL. Age > 65 years, high serum lactate dehydrogenase (LDH) activity, ECOG performance status ≥ 2, Ann Arbor stage III or IV for anatomical staging, and the number of involved extranodal sites are all components of this index before the use of rituximab to predict DLBCL survival [2, 3]. Although IPI is a reliable prognostic tool, it cannot foretell outcomes in patients with aggressive disease, for whom the three‐year event‐free survival rate is only about 50%. The fundamental biological disparity needs to be addressed [2].

The usefulness of canine DLBCL as a natural simulation for human DLBCL has been demonstrated by several studies [4, 5, 6], making it the most studied tumor in veterinary medicine to date [4]. Prognostic variables for dogs with canine DLBCL are poorly depicted [7]. Poorer prognoses were seen in dogs with DLBCL when certain factors were present, such as thrombocytopenia at diagnosis, higher baseline serum globulin concentration, higher baseline neutrophil count, and older age at diagnosis [8]. Tumor mitotic counts correlate with disease progression in dogs with DLBCL. Dogs with < 20 mitoses per 400 field had a median survival duration of 188 days, while those with more than 20 mitoses per 400 field had a median survival time of 31 days [9]. The percentage of neoplastic cells infiltrating the bone marrow (BM) may also predict survival and time to progression [4, 10]. A correlation was also found between canine systemic inflammatory response and decreased survival time. The inflammatory biomarker C‐reactive protein (CRP) was also linked to decreased progression‐free survival (PFS) [7].

The absence of definitive prognostic markers in canine DLBCL cases has prompted the need to investigate new biomarkers that might aid in detecting the prognosis. We aimed to investigate the prognostic significance of miRNA expression profiles in dogs with DLBCL by comparing miRNA signatures between cases with extremely short and long progression‐free survival. MicroRNAs (miRNAs) are small non‐coding RNA molecules that regulate gene expression. They are involved in several pathophysiological processes, including the growth and progression of cancer [11, 12, 13, 14]. Here, we are investigating the potential role of miRNAs as biomarkers in detecting canine DLBCL. This retrospective study with 44 archived lymph node samples of DLBCL‐affected dogs investigated the relationship between miRNA expression levels and PFS and/or best overall response (BOR) to chemotherapy. A group of differentially expressed miRNAs were identified to be linked with short PFS of dogs after chemotherapy treatment. In the future, these results may lead to models to predict treatment outcomes and help tailor treatment plans based on individualized risk assessment.

## Materials and methods

### Animal samples

#### Discovery phase

Six fresh‐frozen (FF) lymph node biopsy specimens collected from dogs with DLBCL were selected for preliminary analysis (Table S1) due to their superior RNA quality over formalin‐fixed paraffin‐embedded (FFPE) samples, which is crucial for accurate small RNA sequencing. All lymph node biopsy samples were collected prior to the dogs receiving any treatment for their DLBCL. Dogs with exceptionally short (*n* = 3) and long (*n* = 3) PFS were specifically selected for this initial study to identify distinct miRNA signatures associated with these divergent outcomes. Progression‐free survival was defined as the time in days from initiation of chemotherapy until cancer progression or death from any cause occurred. After total RNA extraction from the FF lymph nodes, small RNA (sRNA) sequencing proceeded. Several studies have demonstrated comparable results between FF and FFPE samples in various molecular analyses, including gene expression profiling [15, 16, 17] and mutation detection [18], FFPE samples were used to validate the sRNA sequencing results due to their availability in a bigger well‐documented cohort.

#### Validation phase

**Fig. 1:**
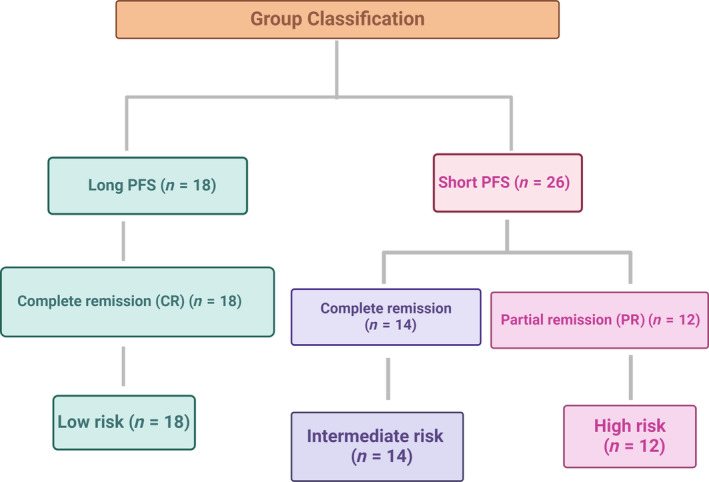
Sample classification for the validation phase (*n* = 44).

Forty‐four FFPE archival lymph node biopsy specimens from dogs with DLBCL were selected to represent two phenotypic groups based on their survival times with known PFS; long PFS (L‐PFS, *n* = 18) and short PFS (S‐PFS, *n* = 26) and three phenotypic groups based on the clinical BOR classifications. The best overall response was classified according to established criteria [19]. Complete remission (CR) was defined as complete regression of all tumor lesions, with resolution of all cancer‐related clinical signs. Partial remission (PR) was defined as a reduction in the sum of the longest diameters of up to five tumor lesions by at least 50% but < 100%. Dogs that experienced partial remission (PR group *n* = 12) had universally short PFS times. Some who achieved complete remission still showed relatively short survival times (CR‐short PFS group *n* = 14), while others showed much longer PFS (Table S2). Combining the PFS and BOR for sample identification, samples with complete remission with long PFS were called low risk. Samples with complete remission with short survival were called intermediate risk, while samples that received partial remission with short survival were renamed to the high‐risk group (Fig. 1). This criterion was used to validate the sRNA sequencing using qPCR. Both FF and FFPE samples were obtained from privately owned dogs diagnosed with primary nodal DLBCL and presented to the Purdue University Veterinary Teaching Hospital (PUVTH), West Lafayette, IN, between 2006 and 2016.

All samples were obtained by surgical biopsy from peripheral lymph nodes (incisional wedge biopsy or surgical extirpation of an entire lymph node) with the dogs under general anesthesia. The diagnosis of DLBCL was determined by combining histopathology and immunohistochemistry findings confirming the expression of CD79a and/or Pax‐5 and the absence of CD3 expression in malignant cells. The demographic information, medical history, and animal test results are provided (Tables S1 and S2). A board‐certified veterinary pathologist evaluated and classified the samples according to the 2008 WHO criteria. In addition to surgical lymph node biopsy, all dogs underwent clinical staging tests, including complete blood count, serum biochemistry profile, thoracic and abdominal radiography, abdominal ultrasonography, and bone marrow aspirate cytology. All dogs were assigned a clinical stage according to WHO criteria [20]. Following staging, all dogs were treated with an identical chemotherapy protocol [21] incorporating cyclophosphamide, doxorubicin, vincristine, and prednisone (i.e., CHOP). All samples for this study were collected with the owners' written informed consent, and sample collection procedures were approved by the Purdue Animal Care and Use Committee (protocols #1708001607 and #1111000308).

### Total RNA extraction

The sRNA sequence samples were extracted from fresh‐frozen specimens using Monarch® Total RNA Miniprep Kit (NEB, Ipswich, MA, USA). For the archived FFPE samples, total RNA was extracted from one 20‐mm scroll from the paraffin blocks using the miRNeasy FFPE kit (QIAGEN Inc, Germantown, MD, USA) according to the manufacturer's specifications. The quantity and quality of total RNA were assessed by UV® spectrophotometry (NanoDrop; ThermoFisher Scientific, Waltham, MA, USA). The quantification of miRNA was assisted by a spectrofluorometer (Qubit 4 Fluorometer; ThermoFisher Scientific) using Invitrogen™ Qubit™ microRNA Assay Kits (ThermoFisher Scientific) as demonstrated (Table S3A,B).

### Small RNA library preparation

The small RNA library preparation was guided using the sequencing libraries generated using NEBNext® Multiplex Small RNA Library Prep Set (NEB) for Illumina®. The RNA was subjected to size selection ranging from 15 to 40 nucleotides in length for small RNA. Ligation of adapters at both ends of the small RNA molecules was performed, followed by reverse transcription to produce complementary DNA (cDNA) using M‐MuLV Reverse Transcriptase (RNase H−). Subsequently, the polymerase chain reaction (PCR) amplification was employed using LongAmp Taq 2X Master Mix, SR Primer for Illumina, and index (X) primer that were designed to target the ligated adapters specifically.

### Sequencing and data processing

The prepared small RNA libraries were subjected to high‐throughput sequencing for Illumina® (Novogene Co., Ltd., Beijing, China). The sequencing was performed according to the manufacturer's protocols to generate raw sequencing reads. The proportion of reads exhibiting < 1% error rate (Q20) and 0.1% error rate (Q30), as well as their GC‐content, was determined after the removal of reads containing poly‐N, 5′ adapter contaminants without 3′ adapter or the insert tag. Additionally, reads containing poly A, T, G, C, or those with low quality were excluded from the analysis, as indicated.

### Read mapping and annotation

MiRNAs were counted using mirdeep2, version 2.0.1.2 [22] with the *Canis lupus familiaris* CanFam 3.1 genome [23] as the reference sequence. For the assignment of miR names, reference lists of dog and mouse mature and precursors were downloaded from miRbase [24] 4.35 Mreads were examined, and 445 known (276) and novel (169) miRNAs with read count > 330 (0.00001%, summed across all samples) identified. These reads account for 39.3 Mreads (90.6%) of the original counts. miR with identical mature sequences (which cannot be distinguished in the sequencing) were merged, resulting in 373 observed miRs (224 known, 149 novel). Normalized counts were determined using the median of ratios method implemented in deseq2 [25]. Predicted miRNA (novel miRNA) that match neither the known dog nor mouse miR lists are assigned names by miRDeep2 that consist of the chromosome number followed by the beginning position of the miRNA in the reference genome sequence, for example, miRNA 12_21493 is found on chromosome 12 at base 21493 (Table S4).

### Differential expression analysis

Differential expression analysis of small RNAs was performed using deseq2 for dog patients with different PFS, S‐PFS, *n* = 3 and L‐PFS, *n* = 3. Small RNAs with a significant fold change of > 3 and adjusted *P*‐value < 0.01 were used as the threshold for significantly differential expression.

### Quantitative RT‐PCR

The 44 FFPE archival lymph node biopsy specimens from dogs with DLBCL were used to extract total RNA. Following the manufacturer's protocol, the first‐strand cDNA synthesis was performed using the miRCURY LNA RT Kit (QIAGEN). The cDNA was diluted at 1 : 80 according to the manufacturer's instructions. Quantitative RT‐PCR was performed on a QuantStudio3 PCR system (ThermoFisher Scientific). The miRCURY LNA [26] miRNA Custom PCR Panel (Cat #: YCA38192; QIAGEN) was used to quantify the pre‐selected miRNAs based on the differential expressed miRNAs between short and long PFS groups in the discovery phase using FF samples showed potential prognostic value canine lymphoma and previous literature on miRNAs role in cancer prognosis [27, 28, 29, 30, 31, 32, 33, 34] (Table 1). Two samples were measured per plate in duplicate for 22 miRNAs, UniSp6 (cDNA synthesis control), UniSp3 (RT‐qPCR positive control) and inter‐plate calibrator (IPC), and non‐template control (negative control). Relative miRNA expression levels were calculated by the $2^{\left(-\Delta \Delta C_{q}\right)}$ method [35].

**Table 1:** The selected miRNAs for miRNA custom PCR panel.

miRNA ID	miRCURY assay cat. no.	Sequence
hsa‐miR‐361‐5p	YP00206054	UUAUCAGAAUCUCCAGGGGUAC
cfa‐miR‐101	YP00205955	UACAGUACUGUGAUAACUGA
hsa‐miR‐29c‐3p	YP00204729	UAGCACCAUUUGAAAUCGGUUA
hsa‐miR‐127‐3p	YP00204048	UCGGAUCCGUCUGAGCUUGGCU
hsa‐miR‐128‐3p	YP00205995	UCACAGUGAACCGGUCUCUUU
hsa‐miR‐129‐5p	YP00204534	CUUUUUGCGGUCUGGGCUUGC
hsa‐miR‐130b‐3p	YP00204317	CAGUGCAAUGAUGAAAGGGCAU
hsa‐miR‐152‐3p	YP00204294	UCAGUGCAUGACAGAACUUGG
cfa‐miR‐1840	YP02102662	UCACGUGACGGGCCUCGGCG
hsa‐miR‐187‐3p	YP00204018	UCGUGUCUUGUGUUGCAGCCGG
hsa‐miR‐192‐5p	YP00204099	CUGACCUAUGAAUUGACAGCC
cfa‐miR‐196b	YP02113947	UAGGUAGUUUCCUGUUGUUGGGA
cfa‐miR‐23a	YP00205956	AUCACAUUGCCAGGGAUUU
cfa‐miR‐889	YP02121125	UUAAUAUCGGACAACCAUUGUU
cfa‐miR‐411	YP02114619	AUAGUAGACCGUAUAGCGUACG
bta‐miR‐99a‐5p	YP00205945	AACCCGUAGAUCCGAUCUUGU
hsa‐miR‐381‐3p	YP00205887	UAUACAAGGGCAAGCUCUCUGU
hsa‐miR‐590‐3p	YP00205448	UAAUUUUAUGUAUAAGCUAGU
cfa‐miR‐125a	YP02113289	UCCCUGAGACCCUUUAACCUGU
bta‐miR‐195	YP00205969	UAGCAGCACAGAAAUAUUGGCA
hsa‐miR‐9‐5p	YP00204513	UCUUUGGUUAUCUAGCUGUAUGA
hsa‐miR‐16‐5p	YP00205702	UAGCAGCACGUAAAUAUUGGCG

### Quantitative RT‐PCR data analysis

Data analyses were performed in r, version 4.2.2 [36]. Relationships between survival times and demographics (Table 2) and clinical variables (age, sex, and cancer stage and grade at diagnosis) were analyzed by multiple regression considering 25 potential models that included all possible additive and interaction effects. Intact and neutered subjects were grouped by sex due to the small number of unneutered subjects in the sample. Differences in mean miRNA expression among the three phenotypic groups were analyzed by Welch's one‐way ANOVA for unequal variances with Bonferroni‐Holm‐corrected pairwise comparisons. In addition to the pairwise comparisons, we examined the linear contrast between the low‐risk group (with long survival) and the pooled intermediate‐ and high‐risk groups (short survival). Effect sizes for all contrasts were quantified using Cohen's *d*, standardized to the average variance of the groups being compared.

**Table 2:** Groups number and survival period in days.

Group	*n*	Progression‐free			
		Mean	SD	Min.	Max.
Short	26	64.3	23.0	19	103
BOR = PR	12	48.5	16.3	19	75
BOR = CR	14	76.2	20.3	35	103
Long	16	460.8	107.2	343	684
Very long	2	1043.0	35.4	1018	1068

Multiple regression and Cox proportional hazards regression were used to investigate relationships between survival time, miRNA expression levels, and demographic and clinical variables. Due to the large number of potential explanatory variables, we used lasso feature selection in the glmnet package [37, 38] to identify the collection of predictor variables most useful for survival prediction. We used 10‐fold cross‐validation (CV) and selected the penalty weight, lambda, that yielded the simplest model with a predictive error within 1 standard error of the minimum (i.e., “lambda.1se”), as recommended by Friedman *et al*. [37].

Lasso selection was also used to construct a multinomial logistic regression (MLR) model to predict patient phenotypes. Performance was assessed by calculating the overall classification error rate in a 10‐fold CV. In addition, because cross‐validation results depend on the random assignment of subjects to folds, we repeated the cross‐validation analysis 200 times to assess the stability of its predictions and variable selections. miRNA expression levels were log_2_ transformed before their incorporation into the models.

We developed a multinomial logistic regression model to classify individual subjects as low, medium, or high‐risk based on miRNA expression levels. A subset of predictive miRNAs was identified by LASSO using 200 replicated 10‐fold cross‐validation (CV) runs. Within each run, the subjects were randomly divided into 10 groups. Then, the model was fitted to the pooled data from 9 groups, and the resulting fit was used to classify the subjects in the 10th group. This was repeated 10 times to predict each group, and then the results were evaluated at lambda.1se to obtain a final model. For details on LASSO model fitting procedures, including the lambda.1se criterion [37]. Cross‐validation results are only partly determined by model performance *per se*. They also depend on the training and test data sets used in the validation process. By replicating the procedure described above, we could evaluate the model's sensitivity to such sampling effects and its predictive performance for individual subjects. Predictions from a stable model (i.e., one that is not overly dependent on the specific dataset to which it is fit) should be consistent across CV runs, regardless of the predictions' correctness. Ideally, the model should classify each subject the same way every time, irrespective of the data on which it was trained. Conversely, a sensitive model may produce very different predictions if trained on a different dataset.

## Results

### RNA extraction and small RNA sequencing

The mean concentration of the total RNA extracted was 1390.47 ± 918.83 ng·μL^−1^ as measured by UV spectrophotometry. The 260/280 nm absorbance ratio was 2.1 ± 0.01 (Table S3). Total: 39.3 million raw reads were obtained from the small RNA libraries generated from fresh frozen lymph nodes. After quality control and filtering, (90.6%) of clean reads were retained for further analysis. A comprehensive overview of the cleaning procedure provides an analysis of the mapping statistics about the reads (Table S4).

### Differential expression analysis of the small RNA sequencing data

**Fig. 2:**
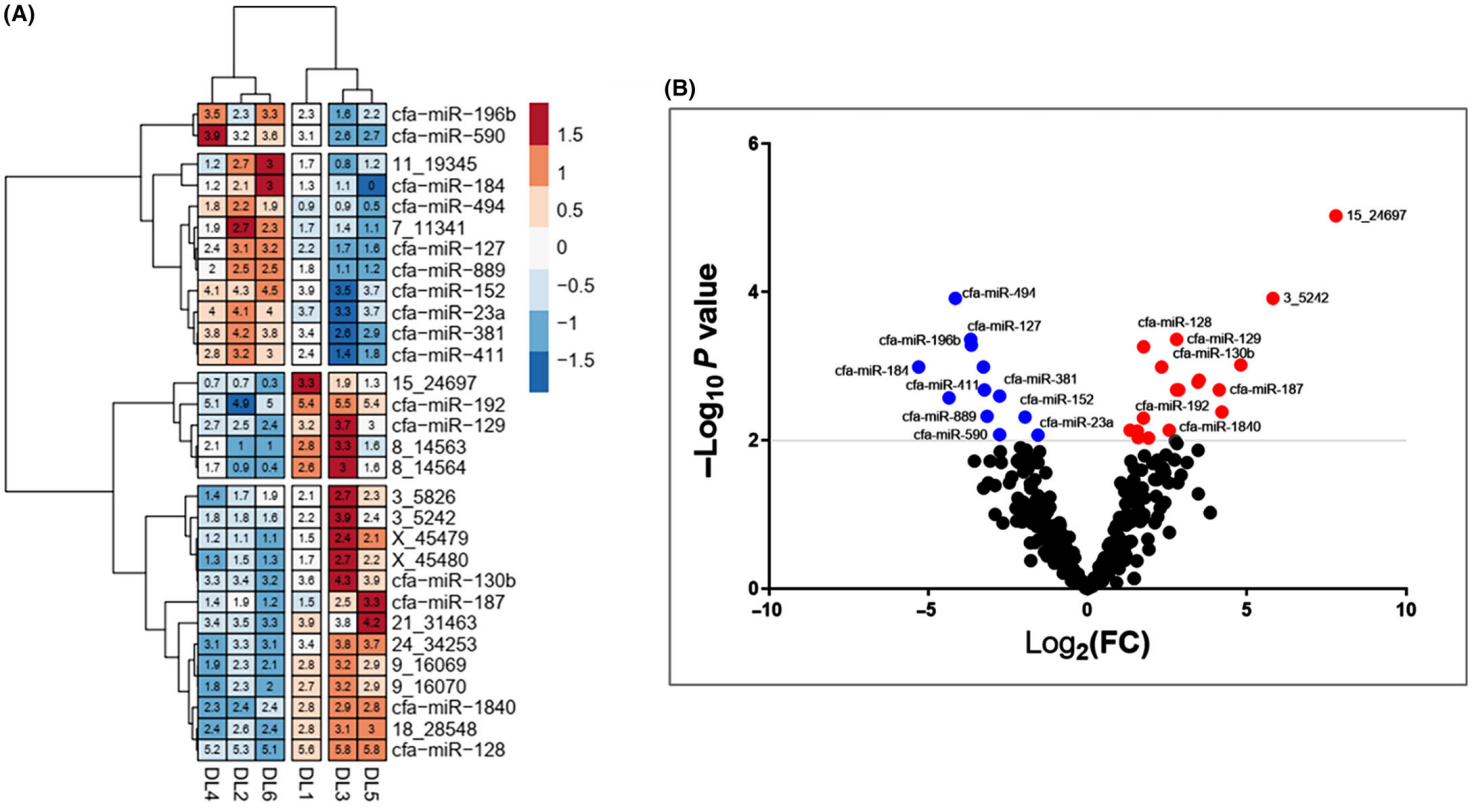
(A) Small RNA sequencing (sRNA‐Seq) heatmap of the differentially expressed miRNAs (DEMs) associated with progression‐free survival (PFS) in dogs with diffuse large B‐cell lymphoma (DLBCL). Red represents high expression; blue represents low expression. The color intensity from red to blue represents the log‐expression values for each miRNA when converted to standard normal deviation (*Z* scores). The samples with short PFS are DLBCL1,3,5 (*n* = 3) and those with DLBCL with long PFS survival are DLBCL 2,4,6 (*n* = 3). The miRNAs with greater than three‐fold expression differences (i.e., log FC > 1.58) and adjusted *P*‐values < 0.01 were selected. (B) The small RNA‐seq volcano plot of the differentially expressed miRNAs (DEMs). Red represents high expression; blue represents low expression. The miRNAs with greater than three‐fold expression differences (log_2_FC > 1.58) and (−log_10_ adj *P*‐values < 0.01 = 2).

Differential expression analysis revealed 30 differentially expressed small RNAs between DLBCL dog patients with different PFS, S‐PFS, *n* = 3 and L‐PFS, *n* = 3, and 18 miRNAs are upregulated. In contrast, 12 miRNAs are downregulated (fold change > 3 and adjusted *P*‐value < 0.01). The results are depicted in a heat map (Fig. 2A) and a volcano blot (Fig. 2B). The heatmap shows the log of the normalized expression of the selected miRNAs clustered according to correlation across samples using Ward's clustering method. Before clustering, the log‐expression values for each miRNA are converted to standard normal deviation (*Z* scores). The normalization method used is *Z*‐score normalization. Transforming the log‐expression values for each miRNA to have a mean of zero and a standard deviation of one ensures that the miRNA expression values are standardized and comparable across samples, allowing for accurate clustering and analysis of differential expression miRNA. The samples with short PFS are DLBCL1,3,5, and those with DLBCL with long PFS survival are DLBCL 2,4,6. SRNA‐seq showed over‐expressions of miR‐187‐3p, miR‐192‐5p, miR‐128, and miR‐1840 miRNAs (18_28548, 21_31463, 24_34253, 9_16069, and 9_16070) in the short PFS group (DL 1,3,5), while miR‐23a, miR‐494, miR‐381, miR‐411, miR‐152, and miR‐196b are overexpressed in the long PFS group (DL 2,4,6).

### Quantitative PCR analysis

The UniSp6 cDNA synthesis control was amplified in all samples at 18.4 ± 1.1 cycle quantification (*C*_q_) (Table S5). UniSp3, a positive qPCR control and inter‐plate calibrator was amplified in all samples, and *C*_q_s were calibrated to 21.64 *C*_q_ across the board to reduce variability from one plate to another. Welch's 2‐sample *t*‐test was used to compare the relative expression of each miRNA in each sample to the relative expression level of miR‐361‐5p (Table 3) based on our previous research [39]. Normalizing miRNA data is complicated and uses diversifiable methods such as exogenous spike control [40], geometrical mean of all miRNAs expressed in one sample [41], and endogenous single or multiple miRNAs [42]. Pradervand *et al*. [43] underscored the importance of efficient miRNA data normalization for miRNA expression quantitation error reduction and controlling experimental technical variations.

**Table 3:** Differences in mean miRNA expression among three phenotypic groups based on progression‐free survival (PFS) and clinical best overall response (BOR) classifications. Dogs were in the high‐risk (partial remission group *n* = 12), complete remission with low survival (intermediate‐risk group *n* = 14), and complete remission with high survival (low‐risk group *n* = 18) in 44 canine lymphoma cases.

miRNA	df	*F* ^a^	Raw *P*	FDR *P*	Low risk vs other^b^		Low vs high‐risk		Low vs intermediate		Intermediate vs low risk	
					*d* ^c^	*P* ^d^	*d*	*P*	*d*	*P*	*d*	*P*
hsa‐miR‐192‐5p	2, 22.9	7.809	0.0026	**0.0299**	−1.184	**0.001**	−1.03	**0.032**	−1.301	**0.005**	0.15	0.708
hsa‐miR‐16‐5p	2, 25.4	7.521	0.0027	**0.0299**	−1.137	**0.002**	−1.25	**0.005**	−1.107	**0.012**	0.325	0.407
hsa‐miR‐29c‐3p	2, 19.1	5.274	0.015	0.0886	0.4	0.336	−0.22	0.595	1.179	**0.021**	−0.953	0.08
cfa‐miR‐196b	2, 23.5	4.945	0.0161	0.0886	−0.531	0.268	−0.14	0.724	−1.05	**0.021**	0.682	0.268
cfa‐miR‐101	2, 22.1	4.401	0.0246	0.0902	−0.774	**0.036**	−0.56	0.358	−1.047	**0.03**	0.16	0.695
bta‐miR‐99a‐5p	2, 23.5	4.224	0.0271	0.0902	−0.631	0.079	−1.13	**0.038**	−0.177	0.624	−0.988	0.07
hsa‐miR‐590‐3p	2, 23.7	4.138	0.0287	0.0902	−0.651	0.12	−0.36	0.644	−1.009	**0.028**	0.412	0.644
hsa‐miR‐152‐3p	2, 24.6	3.624	0.0417	0.1045	−0.804	**0.046**	−0.96	0.058	−0.661	0.154	−0.292	0.466
bta‐miR‐195	2, 26	3.568	0.0427	0.1045	−0.839	**0.041**	−0.87	0.079	−0.799	0.079	−0.05	0.9
cfa‐miR‐125a	2, 24.5	3.428	0.0487	0.1072	−0.808	**0.046**	−0.9	0.085	−0.712	0.106	−0.335	0.412
cfa‐miR‐889	2, 25.2	2.993	0.0682	0.1364	−0.755	0.077	−0.68	0.168	−0.816	0.084	0.059	0.884
hsa‐miR‐187‐3p	2, 26.1	2.839	0.0766	0.1405	−0.657	0.132	−0.88	0.092	−0.459	0.384	−0.533	0.384
hsa‐miR‐130b‐3p	2, 25.6	2.672	0.0883	0.1495	−0.398	0.449	−0.7	0.189	−0.135	0.696	−0.84	0.189
cfa‐miR‐23a	2, 23	2.383	0.1147	0.1802	−0.318	0.572	−0.75	0.223	0.193	0.592	−0.888	0.169
hsa‐miR‐127‐3p	2, 24.3	2.129	0.1406	0.2061	−0.639	0.166	−0.64	0.263	−0.631	0.263	−0.087	0.828
hsa‐miR‐129‐5p	2, 26.8	1.838	0.1787	0.2457	−0.6	0.259	−0.5	0.33	−0.671	0.259	0.305	0.439
hsa‐miR‐9‐5p	2, 26.8	1.56	0.2286	0.2903	−0.191	1	−0.52	0.555	0.05	1	−0.595	0.555
hsa‐miR‐361‐5p	2, 22	1.536	0.2375	0.2903	−0.245	0.815	0.057	0.888	−0.615	0.407	0.473	0.758
hsa‐miR‐128‐3p	2, 23.6	1.389	0.2691	0.3116	0.17	1	−0.18	1	0.507	0.592	−0.595	0.592
hsa‐miR‐381‐3p	2, 24.7	1.27	0.2984	0.3282	−0.408	0.585	−0.61	0.489	−0.211	0.585	−0.491	0.585
cfa‐miR‐411	2, 24.5	1.086	0.3534	0.3702	−0.451	0.589	−0.41	0.589	−0.481	0.589	−0.01	0.98
cfa‐miR‐1840	2, 24.8	0.953	0.3991	0.3991	−0.413	0.737	−0.34	0.751	−0.474	0.737	0.078	0.847

^a^ Welch's one‐way ANOVA with unequal variances.

^b^ “Other” pools intermediate risk and high risk.

^c^ Cohen's *d* using the average variance of the contrasted groups.

^d^ Bonferroni‐Holm adjusted *P*‐value for contrast in the group means.

Values in bold are statistically significant.

#### Survival patterns

The phenotype groups showed distinct survival patterns (Fig. 2 and Table 2). Dogs in the high and intermediate‐risk groups had rapidly progressing diseases with short PFS (≤ 103 days). Within these two groups, dogs in an intermediate risk have longer average survival rates than those in partial remission (Welch's *t*‐test, PFS: *t* = 3.855, df = 23.9, *P* = 0.0008). However, the low‐risk group had longer times for PFS (≥ 343 days). Two dogs in the low‐risk group belonged to a potential fourth phenotype, with PFS > 2 years. However, because this group had only two members, it was retained as part of the low‐risk group for analyses. Thus, the dogs at low risk not only had better PFS but also survived for longer periods after their cancer relapsed.

#### Demographic and clinical relationships with survival

We found few relationships between survival and clinical or demographic variables. Of the 25 models that we considered, only 2 had significant global *F*‐tests (Age: *F* = 4.305, df = 1 and 42, *P* = 0.0442; Age + Grade: *F* = 3.237, df = 3 and 40, *P* = 0.0321). The best‐performing model included additive effects for age and grade at diagnosis. Unsurprisingly, older dogs and those with intermediate‐ or high‐grade cancers did not fare as well as younger animals or those with low‐grade cancers. However, the model only accounted for a small portion of the variation in PFS (adj. *R* ^2^ = 0.135), and the decision to include grade in addition to age yielded only a marginal improvement over the Age‐only model.

#### Differences in miRNA expression among phenotypes

**Fig. 3:**
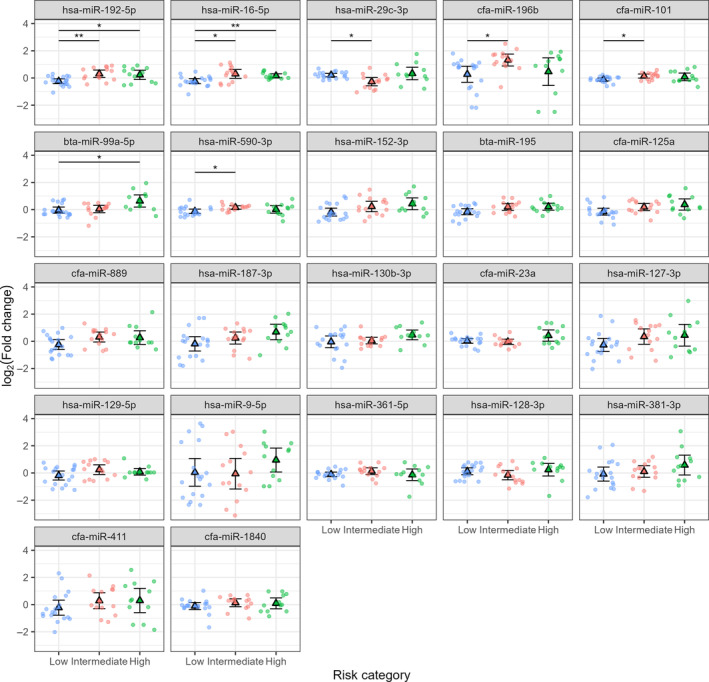
Expression of 22 miRNAs in three phenotypes of canine lymphoma using Welch's one‐way ANOVA for unequal variances with Bonferroni‐Holm‐corrected pairwise comparisons. Dogs in the Low‐risk group (*n* = 18) were clinically classified as being in complete remission and had relatively long progression‐free. The intermediate‐risk group (*n* = 14) also appeared to be in complete remission but had shorter survival rates, more similar to the high‐risk (partial remission) group (*n* = 12). Triangles represent group means ± 95% confidence intervals (CI). The high‐risk, partial remission group; intermediate risk, complete remission with short survival group; Low risk, complete remission with long survival group. *Significant at *P* < 0.05; **Significant at *P* < 0.01.

After FDR correction, only two markers, miR‐192‐5p and miR‐16‐5p, showed significant differences (*P* < 0.05) in mean expression in one‐way ANOVA tests. On average, both miRNAs were expressed at lower levels in the low‐risk phenotype than in the intermediate‐risk or high‐risk group. However, the values for Cohen's *d* (Table 3) group means were generally separated by ≤ 1.3 SD. Thus, although the group averages appear to differ, individual subjects show considerable overlap among groups for all miRNAs. It is worth noting that several additional markers had adjusted ANOVA *P*‐values < 0.1 and had Bonferroni‐Holm corrected *P* < 0.05 for at least one contrast. These included miR‐29c‐3p, miR‐196b, miR‐101, miR‐99a‐5p, miR590‐3p, miR‐195, miR‐152‐3p, and miR‐125a. Given the small size of the current sample, these markers may warrant additional future investigation. They should also be considered in potential multivariate classifiers. The miRNAs differential expression data analysis results are summarized in Table 3 and illustrated in Fig. 3.

#### Prediction of survival time

We attempted to predict survival times using multiple regression and Cox models using lasso feature selection. Using linear regression, the lasso selected only one predictor, miR‐192‐5p, which yielded an *R* ^2^ = 0.253 for the relaxed model. Consistent with Fig. 3, survival times declined with increasing expression of miR‐192‐5p (*t* = −3.769, df = 42, *P* = 0.0005). However, analysis of the model residuals showed that it consistently predicted survival times within a narrow range from 124 to 165 days. In contrast to multiple regression, the Cox model selected three predictors: miR‐192‐5p, miR‐16‐5p, and miR‐152‐3p. All three miRNAs were positively related to the hazard ratio (consistent with Fig. 3).

#### Phenotype classification

Given the high levels of overlap among group‐wise miRNA expressions (Fig. 3), none of the miRNAs studied here can serve as a useful prognostic measure to distinguish low‐risk from intermediate‐risk or high‐risk cases on its own. Therefore, we used multinomial logistic regression with lasso feature selection to explore potential multivariate prognostic indices.

Multinomial logistic regression (MLR) generalizes the methodology of binary logistic regression to categorical responses with > 2 potential classes of outcomes. Unlike more familiar forms of the generalized linear model (GLM), which predict outcomes based on a single linear predictor, MLR generates a linear predictor for each possible outcome. In a classification setting, new observations are typically assigned to the class whose predictor has the largest value. In our analysis, miRNAs miR‐192‐5p and miR‐16‐5p consistently loaded onto the predictor for low risk (Figs S1) for cross‐validation results. Based on these variables, the model correctly classified all 18 members of the low‐risk group and correctly classified half of the members of the intermediate‐risk and high‐risk groups. In most cases, misclassified individuals were placed at low risk (Table 4). Individual classifications in the low‐risk and intermediate‐risk groups were generally consistent across CV runs (i.e., individuals were almost always classified similarly; Fig. S1). In contrast, classifications for six high‐risk group members varied among CV runs.

**Table 4:** Confusion matrix for prediction of canine lymphoma phenotype using multinomial logistic regression and lasso feature selection, averaging over 200 cross‐validation sets.

Truth	Prediction		
	Low risk	Intermediate risk	High risk
Low risk	18	0	0
Intermediate risk	7	7	0
High risk	5	1	6

## Discussion

Despite receiving standardized CHOP chemotherapy treatment, dogs with DLBCL demonstrate significant heterogeneity in terms of survival outcomes [8, 9]. While the multidrug regime of chemotherapy CHOP has demonstrated promising effectiveness in certain dogs with DLBCL, a significant proportion of cases fail to attain complete remission or relapse following a short period of remission, contributing to poor outcomes and a significant mortality rate due to this disease. Clinical studies suggest that a subgroup of dogs with DLBCL derives only a slight benefit from CHOP chemotherapy. To date, this subpopulation is not easily identified with current prognostic variables. In human studies, the International Prognostic Index (IPI) scoring system utilizing different clinical factors, including age, disease stage, number of extranodal sites, and performance status, is used to sort patients into different risk groups. IPI is still in use after revision, although it fails to represent the cellular morphology heterogeneities precisely and tends to incline to overemphasize the high‐risk patient's percentage [44]. Unlike human studies, limited research studies have been conducted on prognostic factors in dogs with diffuse large B‐cell lymphoma.

MicroRNAs (miRNAs) are diminutive non‐coding ribonucleic acids (RNAs) that control protein synthesis at the post‐transcriptional stage. This short RNA class exhibits high conservation and binding affinity to the 3′ untranslated region of target mRNA through complementary base pairing [45], resulting in the target mRNA degradation or inhibition of protein translation [46]. Many studies have implied the important regulatory role of miRNAs interfacing with pseudogenes and various types of RNAs, including long non‐coding RNA, circular RNAs, and messenger RNAs within human cells [47]. Each microRNA (miRNA) can govern up to hundreds of genes, orchestrating various biological functions, including cell growth, differentiation, proliferation, and apoptosis in addition to pathological conditions such as tumorigenesis, drug resistance, malignant transformation, anchorage‐independent growth, new blood vessel formation, cell growth, differentiation, proliferation, and apoptosis [48]. These effects are accomplished by altering the abundance of downstream target proteins. Therefore, because of its central role in governing cancer phenotypes, miRNA exhibits significant potential as a prognostic indicator and an innovative target for cancer therapeutics [49, 50].

This study investigated miRNAs' signatures as a supplementary prognostic tool. We could differentiate between L‐PFS and S‐PFS groups of the DLBCL cases based on the survival rates using the expression level of two miRNAs: miR‐192‐5p and miR‐16‐5p. The two markers, miR‐192‐5p, and miR‐16‐5p, showed significant expression differences between low‐risk, intermediate, or high‐risk phenotypes. They also separated the short survival groups based on survival rate and BOR as they were expressed at lower levels in intermediate‐risk or high‐risk than low‐risk phenotype. Growing evidence supports the differences in miRNA expression profile observed between malignant cells and their normal counterparts; likewise, these differences correlate with prognostic factors in cancer.

The exact implications of miR‐192 in tumor development are still ambiguous in human studies. For instance, frequent studies investigated the role of miR‐192 in cancer progression and suggested its role as a tumor suppressor miRNA in human bladder cancer [51], pediatric acute lymphoblastic leukemias [52], and human hepatocellular carcinoma, [53] among others. A previous study conducted in our lab using small RNA sequencing (sRNA‐seq) and RT‐qPCR data analysis showed the upregulation of miR‐192‐5p in DLBCL cases compared to healthy groups of dogs [39]. This finding is supported by the significant overexpression of miR‐192‐5p in the low‐grade B‐cell lymphoma, Waldenstrom Macroglobulinemia [54, 55].

MiR‐16‐1 is one of the controversial miRNAs. It is a member of the miR‐15/107 superfamily, which initiates apoptosis [56], is the precursor form of the miRNA miR‐16, which comprises miR‐16‐5p and miR‐16‐3p (depending on the two arms of the precursor hairpin structure, the suffix is either “‐5p” or “‐3p”). Subsequently, both have different functions and target genes regulating different cellular processes owing to discrete sequences, even though they originate from the same precursor. In leukemia, miR‐15a/miR‐16‐1 was the first tested for tumor‐induction [57]. An experiment conducted using cell lines derived from patients with B‐cell chronic lymphocytic leukemia (B‐CLL) revealed that miR‐15a and miR‐16‐1 are deregulated and directly target the p53 protein in a positive feedback loop, wherein these miRNAs are also regulated by direct binding of p53 [57]. Chen and Zhang *et al*. suggested its association with the pathogenesis of lymphoma subtypes, such as mantle cell lymphoma [58, 59]. To the authors' knowledge, no study compares miR‐16‐5p in DLBCL groups with different survival and outcomes. Our study found miR‐16‐5p upregulated in the intermediate risk and high risk compared to low risk. The miR‐17‐5p and miR‐16‐5p were found to have the strongest correlations with specific mRNA targets like B‐cell lymphoma 2 (BCL2) and mothers against decapentaplegic 3 (SMAD3) genes. It is also involved in suppression of cytokine signaling 1 (SOCS1) implicated in cancer metastasis, cell cycle, adherence junctions, and interactions between the extracellular matrix and receptors leading to brain metastases in patients with advanced breast cancer [60]. Furthermore, miR‐16‐5p was detected to be overexpressed in tumor samples from breast cancer compared to healthy subjects after four validation phases following a screening phase [61]. Likewise, miR‐16‐5p showed a drop in its expression in the plasma of patients' post‐surgical removal of colorectal tumor, suggesting the source of the miRNA and the possibility of using it as an early detecting non‐invasive biomarker [62]. These findings align with miR‐16‐5p expression in tumor tissue of colorectal cancer where it is over‐expressed compared to its adjacent tissue [62]. Additionally, Lung cancer [63], esophageal squamous cell carcinoma (ESCC) [64], Kaposi's sarcoma [65], upper tract urothelial carcinoma (UTUC) [66], high‐grade serous ovarian carcinoma tissues [67], and ovarian cancer [68] are all showed overexpression in the cancerous samples compared to the healthy peers. Although there is inter‐individual variation in the expression levels of miRNAs, there is evidence that average miRNA expression differs among the phenotype groups. On the other hand, miRNA‐16 used to be known as endogenous control [69, 70] and key player in diverse malignancies development [71, 72], including neuroblastoma [73], osteosarcoma [74], hepatocellular carcinoma [75], breast cancer [76].

The MLR classifier could only correctly classify half of the intermediate‐risk and high‐risk individuals in the dataset. However, given that PR individuals are readily identified by current clinical methods, and that those methods cannot identify intermediate‐risk individuals, our results still represent a significant improvement. Assuming that the current findings can be validated, miRNA assays might complement existing clinical methods and improve the prognostic assessment of dogs with DLBCL. However, they cannot replace existing methods based on the current results. Thus, these proposed prognostic biomarkers require further investigation in prospective studies and confirmation in a larger dataset.

Despite the relatively small univariate effect sizes we have reported for miRNAs in this study, co‐expression patterns may still provide useful prognostic information. We are currently investigating the possibility that high‐risk and intermediate‐risk phenotypes are characterized not by specific changes in miRNA expression but rather by the presence of any dysregulation that carries them outside a certain range of co‐expression patterns. In other words, high‐risk and intermediate‐risk patients may be identifiable as outliers relative to the multivariate distribution of miRNA expression among low‐risk patients. Similarly, low‐risk cases may represent outliers relative to healthy individuals, but we cannot investigate this possibility with the current data set.

Having a developed prognostic index that integrates multiple biomarkers parallel to the widely utilized IPI in human medicine would represent a valuable progression in this research area. A dependable prognostic index will be significant in assisting pet owners in making well‐informed decisions regarding treatment options for their canine companions. It will also play a crucial role in interpreting and comparing clinical trial outcomes for better innovative therapeutic approaches. Effective prognostic indicators can act as a solid base for patients' stratified treatment; in that way, excessive treatment and unnecessary expenses will be avoided. The identified miRNAs in this study, together with other differentially expressed miRNAs in canine lymphoma, will be included in future studies to elucidate their roles miRNAs in DLBCL pathogenesis.

Although our study provided significant findings, the relatively small sample size of 44 dogs was a limitation for generalizing the findings. A larger cohort with a diverse population, including age, sex, breed, and response to treatment, coupled with progression‐free survival, would enhance the robustness and applicability of the findings. Although many researchers have demonstrated comparable results between FF and FFPE samples in various molecular analyses, others have depicted the potential variability between FF and FFPE samples. In this study, the lack of a larger sample size representing the intermediate‐risk group limited obtaining more data representing this group. Furthermore, investigating the newly discovered miRNA and its role in DLBCL pathogenesis is required in future studies.

## Conclusions

Canine DLBCL is the most predominant form of non‐Hodgkin lymphoma (NHL) observed in dogs and is analogous to the same cancer in humans. Identifying those dogs with DLBCL that will respond most and least favorably to current therapies remains challenging. MicroRNAs can potentially enhance the prognostic assessment of dogs with this disease. This study aimed to correlate miRNA expression and progression‐free survival (PFS) in dogs with diffuse large B‐cell lymphoma (DLBCL). A discrete cluster of miRNAs exhibits a significant negative correlation with progression‐free survival (PFS) in dogs diagnosed with diffuse large B‐cell lymphoma (DLBCL). Dogs exhibiting a reduced progression‐free survival (PFS) demonstrated a statistically significant increase in the levels of miR‐192‐5p and miR‐16‐5p. The expression levels of miR‐192‐5p and miR‐16‐5p were significantly decreased in the complete remission group with a high survival rate. MicroRNA profiling can potentially predict the prognosis of dogs diagnosed with DLBCL. The identification of miRNAs that exert an impact on the progression of canine DLBCL may facilitate the development of personalized therapeutic interventions.

## Conflict of interest

The authors declare no conflict of interest.

### Peer review

The peer review history for this article is available at https://www.webofscience.com/api/gateway/wos/peer‐review/10.1002/2211‐5463.13887.

## Author contributions

NOE, MOC, and APS contributed to conceptualization; NOE and MG contributed to data curation; NOE, NIL, and MG contributed to formal analysis; NOE, MOC, MG, and APS contributed to investigation; NOE, MG, and APS contributed to methodology; APS contributed to project administration; APS and NOE contributed to resources; NOE, MG, and EES contributed to software; MOC and APS contributed to supervision; NOE and EES contributed to validation; NOE, EES, NIL, and APS contributed to writing original draft; NOE, EES, MOC, MG, NIL, and APS contributed to writing, review and editing. All authors have read and agreed to the published version of the manuscript.

## Supporting information


**Fig. S1.** Stability of individual‐level risk classifications for 44 dogs diagnosed with lymphoma based on miRNA expression.

**Table S1.** The clinical information of diffuse large B‐cell lymphoma cases used for small RNA sequencing.
**Table S2.** The clinical information of diffuse large B‐cell lymphoma cases used for qPCR.
**Table S3.** The RNA extraction information of samples used for small RNA sequencing.
**Table S4.** Summary of miRNA data cleaning and filtering steps.
**Table S5.** The calculated ΔΔCT for each miRNA in each sample.

## Data Availability

The datasets used and analyzed in the current study are available from the corresponding authors upon reasonable request.
