# Supplementary material for: MicroRNAs implicated in canine diffuse large B‐cell lymphoma prognosis
Source: FEBS Open Bio. 2024 Sep 1;14(11):1899–913. doi: 10.1002/2211-5463.13887 (PMC11532975; doi:10.1002/2211-5463.13887)
Supplement: Supplementary file 1 — Fig. S1. Stability of individual‐level risk classifications for 44 dogs diagnosed with lymphoma based on miRNA expression. [file FEB4-14-1899-s001.docx]

**Supplementary information**


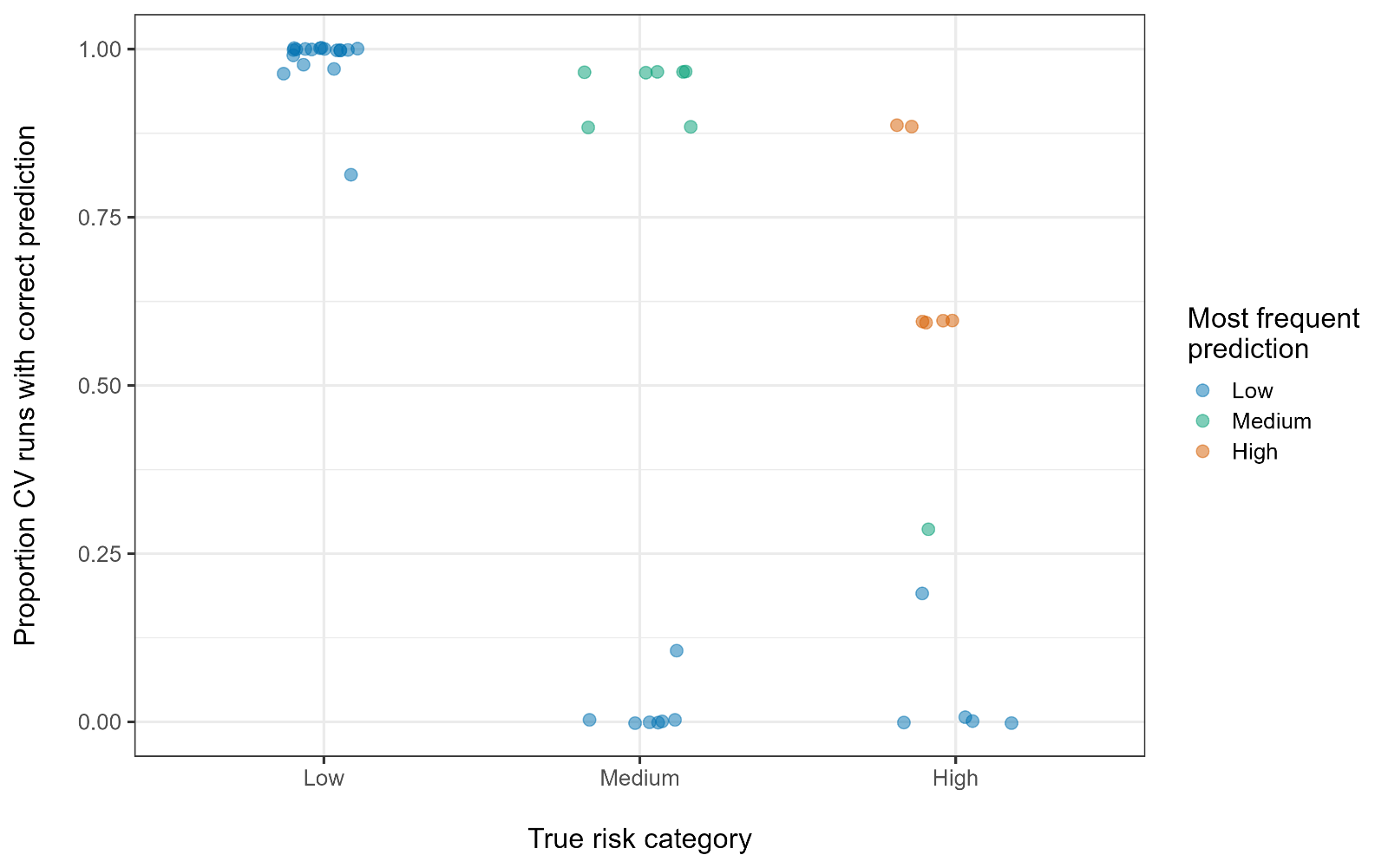


**Supplementary Figure 1**: Stability of individual-level risk classifications for 44 dogs diagnosed with lymphoma based on miRNA expression. Each point represents the proportion of 200 replicate CV-runs in which a single subject was correctly classified. Values near 1 or 0 indicate that the classification result is not sensitive to the selection of training and test sets within the current data (1 = consistently correct prediction, 0 = consistently incorrect). Values near 0.5 indicate that differences in the training data yielded different predictions.
